# Peptone-Induced Physio-Biochemical Modulations Reduce Cadmium Toxicity and Accumulation in Spinach (*Spinacia oleracea* L.)

**DOI:** 10.3390/plants9121806

**Published:** 2020-12-19

**Authors:** Naila Emanuil, Muhammad Sohail Akram, Shafaqat Ali, Mohamed A. El-Esawi, Muhammad Iqbal, Mohammed Nasser Alyemeni

**Affiliations:** 1 Department of Botany, Government College University, Faisalabad 38000, Pakistan; naila60jb@gmail.com (N.E.); iqbalm@gcuf.edu.pk (M.I.); 2 Department of Environmental Sciences and Engineering, Government College University, Faisalabad 38000, Pakistan; 3 Department of Biological Sciences and Technology, China Medical University, Taichung 40402, Taiwan; 4 Botany Department, Faculty of Science, Tanta University, Tanta 31527, Egypt; mohamed.elesawi@science.tanta.edu.eg; 5 Botany and Microbiology Department, College of Science, King Saud University, Riyadh l1451, Saudi Arabia; mnyemeni@ksu.edu.sa

**Keywords:** antioxidants, cadmium, oxidative stress, peptone, spinach

## Abstract

The accumulation of cadmium (Cd) in edible plant parts and fertile lands is a worldwide problem. It negatively influences the growth and productivity of leafy vegetables (e.g., spinach, *Spinacia oleracea* L.), which have a high tendency to radially accumulate Cd. The present study investigated the influences of peptone application on the growth, biomass, chlorophyll content, gas exchange parameters, antioxidant enzymes activity, and Cd content of spinach plants grown under Cd stress. Cd toxicity negatively affected spinach growth, biomass, chlorophyll content, and gas exchange attributes. However, it increased malondialdehyde (MDA), hydrogen peroxide (H_2_O_2_), electrolyte leakage (EL), proline accumulation, ascorbic acid content, Cd content, and activity of antioxidant enzymes such as superoxide dismutase (SOD), peroxidase (POD), and catalase (CAT) in spinach plants. The exogenous foliar application of peptone increased the growth, biomass, chlorophyll content, proline accumulation, and gas exchange attributes of spinach plants. Furthermore, the application of peptone decreased Cd uptake and levels of MDA, H_2_O_2_, and EL in spinach by increasing the activity of antioxidant enzymes. This enhancement in plant growth and photosynthesis might be due to the lower level of Cd accumulation, which in turn decreased the negative impacts of oxidative stress in plant tissues. Taken together, the findings of the study revealed that peptone is a promising plant growth regulator that represents an efficient approach for the phytoremediation of Cd-polluted soils and enhancement of spinach growth, yield, and tolerance under a Cd-dominant environment.

## 1. Introduction

Cadmium (Cd) is a very toxic element and has a high mobility rate from the soil to plants. Cd has been extensively studied in agriculture systems due to its toxic effects on plants and humans [1]. The concentration of Cd is being increased in the soil due to its uses in sludge applications, sewage irrigation, chemical fertilizers, and atmospheric depositions [2,3]. Recently, the contamination of agricultural soils with Cd has become one of the most severe environmental issues in Asian countries [4]. In Pakistan, inorganic fertilizers have been extensively used to increase the yield of crops, resulting in the accumulation of higher concentrations of Cd in the soil. Cd can readily enter into plant roots and translocate to shoots. With increasing the intracellular Cd concentrations, various plant metabolic processes, including cell division, mineral nutrition, nitrogen metabolism, photosynthesis, and respiration, are severely affected [5]. Exposure of plants to Cd stress results in leaf rolling, chlorophyll content decrease, and photosynthesis inhibition via suppressing the chlorophyll biosynthesis [6]. Cd decreases the uptake of micro- and macro-nutrients, which in turn affects the plant transport activities [7]. It also affects nitrate absorption and its translocation from plant roots to shoots via inhibiting nitrate reductase. Moreover, it alters the composition of lipids and causes changes in the membrane functionality. Higher Cd concentrations, within the plant body, generate reactive oxygen species (ROS), which cause oxidative stress [8], leading to the degradation of pigments, nucleic acid, and proteins. The radial absorption capability of Cd by plant roots and its translocation toward the aerial parts make it of great concern regarding its accumulation in the food chain [9]. Vegetables cultivated in contaminated soils accumulate high concentrations of Cd, leading to increases in food contamination risks. Spinach (*Spinacia oleracea* L.) is a leafy vegetable with green and broad large-surface-area leaves, high growth rate, and metal-accumulating ability [10]. Spinach accumulates Cd via plant roots and translocate it to the leaves, resulting in increased food contamination risks. Therefore, there is an urgent need to minimize the accumulation of Cd in spinach plants.

Amino acids are building blocks for protein synthesis and play a critical role in plant metabolism. Amino acids are very important to stimulate cell growth as they can act as a buffer to maintain the optimum pH for plant cells. They eliminate ammonia from plant cells and protect them against ammonia toxicity. They are also involved in the biosynthesis of different organic compounds such as hormones, vitamins, pigments, alkaloids, terpenoids, purine, pyrimidine, and enzymes [11]. Moreover, they are involved in the stimulation of physiological and metabolic functions of plants [12]. Several studies revealed the positive effects of amino acids on plant growth [13,14]. However, very little is known about the amino acids that induce impacts on plants grown under heavy metal stress, particularly Cd.

Biostimulants can regulate natural plant processes to increase the nutrients intake, environmental stress tolerance, and crop yield [15]. It has been suggested that biostimulants could interact with signaling processes and help plants to cope with the environmental stress. Biostimulants are derived from various inorganic and biological compounds, such as fulvic and humic substances, animal feeds, protein hydrolysates, and industrial discards [16,17,18]. Protein hydrolysates increase the activity of enzymes and improve the uptake of nutrients [19]. Hydrolyzed proteins might contain short-chain bioactive peptides, which have immunological and hormonal activities and could act as plant growth regulators [20,21]. Peptone is a mixture of short peptides and amino acids. It is a biostimulant in the form of macro-granules and is produced from the enzymatic hydrolysis of animal or plant proteins. It is mainly involved in the stimulation of plant growth and may provide protection against pathogens. Acid hydrolysate of peptone from vegetable sources is a rich source of the nitrogenous material and contains a high concentration of free amino acids. The optimal concentration of available amino acids can play diverse roles in plant metabolism and growth under stress conditions [22]. Thus, we hypothesized that the physio-biochemical mechanisms of spinach could be significantly regulated through the foliar application of peptone, leading to the alleviation of the harmful impacts of Cd. Therefore, the main objective of the present study was to evaluate the harmful impacts of Cd on spinach growth attributes, biomass, Cd accumulation, and oxidative mechanisms. The present study also examined the possible role of peptone in enhancing the growth and production of spinach plants grown in Cd-contaminated areas via upregulating the defense systems as well as reducing Cd accumulation in edible plant parts.

## 2. Materials and Methods

### 2.1. Soil Analysis

Soil used in the study was taken from agricultural fields and was characterized at the Institute of Soil and Environmental Sciences, University of Agriculture Faisalabad. The soil was cleaned and sieved before using for filling the pots. The methods of Bouyoucos [23] and Page et al. [24] were used for particles size characterization and sodium absorption ratio determination. Soil electrical conductivity (EC) and pH was determined using EC/pH meter. The methodology of Soltanpour [25] was used for extracting the trace elements at pH 7.6 using the ammonium bicarbonate diethylene triamine penta-acetic acid (AB-DTPA) solution. The properties of the soil used for the experiment are shown in Table 1.

### 2.2. Experimental Design

Seeds of spinach (*Spinacia oleracea* L.) were obtained from Ayub Agricultural Research Institute (AARI), Faisalabad. The seeds were sown in plastic pots filled with 5 kg soil/pot. The pots were arranged in a completely randomized design. In order to maintain five seedlings per pot, thinning was performed after 7 days and an appropriate fertilizer was applied. Water polluted with cadmium nitrate (0, 50, and 100 µM) was used for the irrigation of plants. Peptone (Millipore Sigma, 51841) solution (500 and 1000 mg L^−1^) was prepared using ddH_2_O. A 0.5 L peptone solution (or ddH_2_O) was sprayed on 20 plants (four replicates for each treatment with five plants in each pot) twice after an interval of one week. Hence, a plant received 0, 25, or 50 mg of peptone after two spray treatments using 0, 500, and 1000 mg L^−1^ peptone solution, respectively.

### 2.3. Plant Harvesting and Sampling

The plants were harvested after 40 days and separated into shoots and roots to measure the growth parameters. Plants were then washed with a distilled water to remove adhered soil particles and were then air-dried. The plant fresh weight was then measured using an analytical balance. The plants were oven dried at 70 °C for 48 h to be used for estimating the dry weight of root and shoot, discretely.

### 2.4. Determination of Chlorophyll and Gas Exchange Parameters

The method of Arnon [26] was followed to measure the plant chlorophyll content. A 0.5 g leaf sample was dipped in 80% acetone at 4 °C in dark. The reading was then taken using a spectrophotometer at the wavelengths of 645, 663, and 480 nm in order to calculate the chlorophyll content. An IRGA (infra-red gas analyzer) was used to measure the stomatal conductance and photosynthetic rate at a high intensity of light between 10:00 to 12:00 a.m. on a full-sun day.

### 2.5. Determination of Electrolyte Leakage, Hydrogen Peroxide (H_2_O_2_), and Malondialdehyde (MDA)

The protocol of Dionisio-Sese and Tobita [27] was used to measure the electrolyte leakage (EL) level by dipping small pieces of leaves in deionized water. The first EC_1_ reading was taken after the samples’ incubation at 32 °C for 2 h, and the second EC_2_ was determined after the same samples were subsequently incubated at 121 °C for 20 min. The level of EL was calculated as follows:

EL = (EC_1_/EC_2_) × 100

The methods of Velikova et al. [28] and Carmak and Horst [29] were used to measure the levels of MDA and H_2_O_2_ in plants, respectively.

### 2.6. Determination of Superoxide Dismutase (SOD), Peroxidase (POD), and Catalase (CAT) Activity

The method of Zhang [30] was used to determine SOD and POD activities in spinach plants by grinding leaf samples in liquid nitrogen followed by their standardization with 0.5 M phosphate buffer of pH 7.8. The activity of CAT was measured following the protocol of Aebi [31].

### 2.7. Determination of the Contents of Ascorbic Acid and Proline

Ascorbic acid in plant samples was measured following the method of Mukherjee and Choudhuri [32]. The methodology of Bates et al. [33] was used to measure proline content in plant samples.

### 2.8. Determination of Cd Content

Cadmium content in plant samples was measured by digesting 0.5 g of plant samples in HNO_3_ and HCLO_4_ (3:1, *v/v*) in a conical digestion flask using a hot plate (350 °C) for about 8–10 h. The digested samples were run on an atomic absorption spectrophotometer to measure Cd concentration [34].

### 2.9. Statistical Analysis

Statistical analysis for the sample data was performed using SPSS for windows. The results were subjected to the two-way analysis of variance (ANOVA) using Tukey’s test at a probability level of 5%, and the *p* value at 0.05 was considered significant.

## 3. Results

### 3.1. Plant Growth

**Figure 1:**
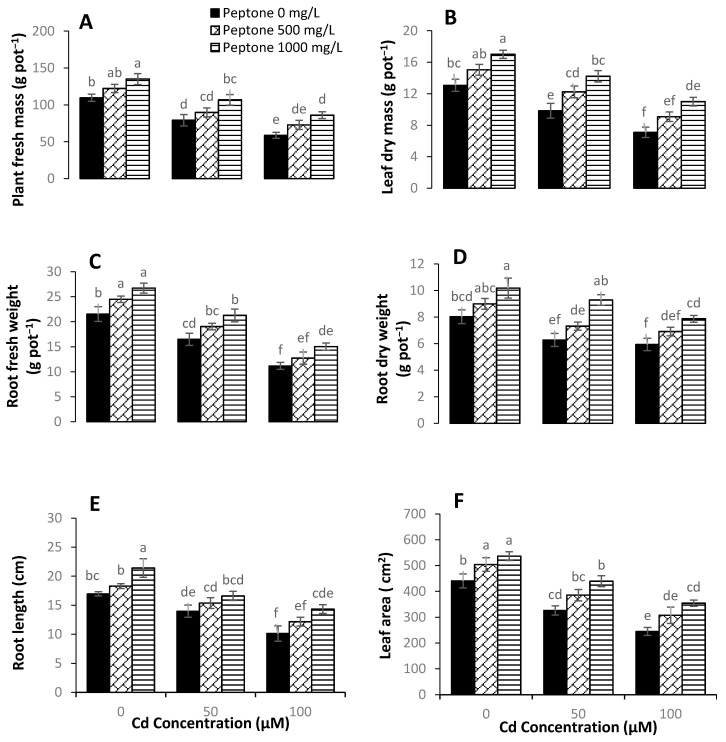
The influence of the foliar application of peptone (0, 500, and 1000 mgL^−1^) on the leaf fresh weight (**A**), leaf dry weight (**B**), root fresh weight (**C**), root dry weight (**D**), taproot length (**E**), and leaf area (**F**) of spinach plants grown under Cd stress. Values represent the means of four replicates with standard deviations. Letters on the bar indicate the significant differences between given treatments at *p* < 0.05.

Plant growth parameters were recorded to measure Cd toxicity in spinach. The results indicated that the foliar application of peptone exhibited positive effects on plant growth and morphology. Under Cd stress, the application of peptone enhanced the plant growth attributes such as fresh and dry biomass, root length and leaf area. By the application of 1000 mgL^−1^ of peptone, the leaf fresh weight exhibited significant increases of 23, 34, and 46% under Cd stress of 0, 50, and 100 µM, respectively, as compared to control. Application of 500 mgL^−1^ of peptone significantly increased the leaf fresh weight at 11%, 13%, and 24% under Cd stress of 0, 50, and 100 µM, respectively, as compared to control (Figure 1A). By the application of 500 mgL^−1^ of peptone, the leaf dry mass was significantly increased up to 15%, 24%, and 27% under Cd stress of 0, 50, and 100 µM, respectively, in comparison to control. A maximum significant increase of 54% in the leaf dry mass was recorded in plants treated with 1000 mgL^−1^ of peptone under 100 µM Cd stress (Figure 1B). The higher level of peptone spray (1000 mgL^−1^) significantly (*p* < 0.05) increased the leaf area up to 21%, 34%, and 44% under Cd stress of 0, 50, and 100 µM, respectively, as compared to control (Figure 1F).

### 3.2. Photosynthetic Pigments

**Figure 2:**
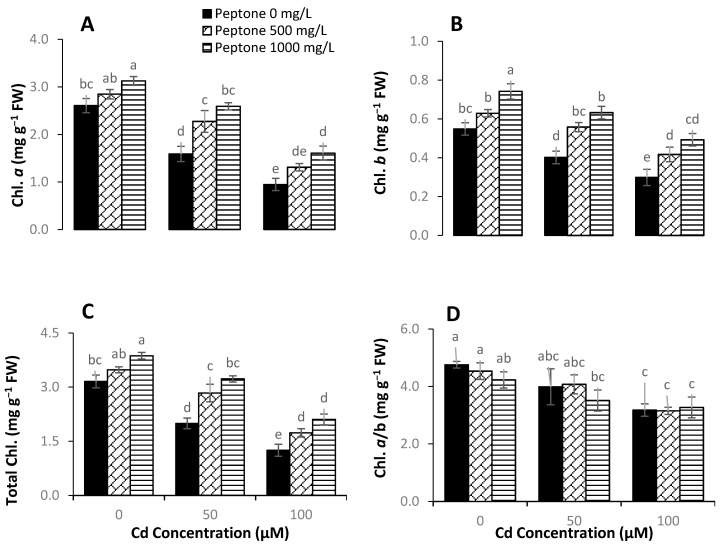
The influence of the foliar application of peptone (0, 500, and 1000 mgL^−1^) on chlorophyll *a* (**A**), chlorophyll *b* (**B**), total chlorophyll (**C**), and chlorophyll *a/b* ratio (**D**) of spinach plants grown under Cd stress. Values represent the means of four replicates with standard deviations. Letters on the bar indicate the significant differences between given treatments at *p* < 0.05.

Photosynthesis is considered a major process involved in plant growth and biomass production. This process is very sensitive to heavy metal uptake/accumulation. Decreases in the activity of the photosynthetic system is pronounced under Cd stress. This might be due to the decreases in chlorophyll biosynthesis and photosynthetic enzymes’ activity as well as the disturbance in the water and nutrient balance in plants. The current study revealed that the application of peptone for Cd-treated and non-treated plants amended the quantity of photosynthetic pigments (Figure 2). Plants treated with peptone (500 mgL^−1^) exhibited a significantly increased chlorophyll *a* content of 9%, 43%, and 38% under Cd stress of 0, 50, and 100 µM), respectively, as compared to control. In comparison with control, a higher significant increase in chlorophyll *a* content of 69% was noted in plants treated with 1000 mgL^−1^ of peptone under 100 µM Cd application (Figure 2A). Plants treated with 1000 mgL^−1^ of peptone significantly increased chlorophyll *b* contents under Cd stress (Figure 2B). Moreover, the exogenous application of 500 mgL^−1^ of peptone significantly enhanced chlorophyll *a/b* ratio under Cd stress in comparison to control. A maximum increase of 11% was recorded in chlorophyll *a/b* ratio of plants treated with 1000 mgL^−1^ of peptone and 100 µM of Cd (Figure 2D).

### 3.3. Gas Exchange Parameters

**Figure 3:**
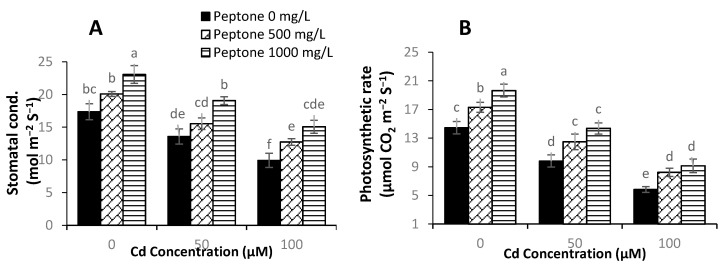
The influence of the foliar application of peptone (0, 500, and 1000 mgL^−1^) on the stomatal conductance (**A**) and photosynthetic rate (**B**) of spinach plants grown under Cd stress. Values represent the means of four replicates with standard deviations. Letters on the bar indicate the significant differences between given treatments at *p* < 0.05.

The plant photosynthetic rate was significantly affected by Cd stress, which inhibits chlorophyll synthesis and results in pigment degradation. Cd stress results in the decrease of the photosynthetic rate through increasing the stomatal resistance and reducing the density of stomata, leading to reductions in the gas exchange rate. In the present study, the application of peptone resulted in the modulation of gas exchange parameters such as the photosynthetic rate and stomatal conductance of spinach plants grown under Cd stress. The photosynthetic rate and stomatal conductance were significantly enhanced upon the foliar application of peptone (Figure 3). Significant increases of 35%, 46%, and 56% in the photosynthetic rate and 32%, 40%, and 51% in the stomatal conductance were recorded in plants treated with 1000 mgL^−1^ of peptone under Cd stress of 0, 50, and 100 µM, respectively. Moreover, the application of 500 mgL^−1^ of peptone significantly increased the photosynthetic rate up to 19%, 27%, and 41% and the stomatal conductance up to 15%, 14%, and 28% under Cd stress of 0, 50, and 100 µM, respectively, as compared to control.

### 3.4. Levels of Hydrogen Peroxide, Malondialdehyde, and Electrolyte Leakage

**Figure 4:**
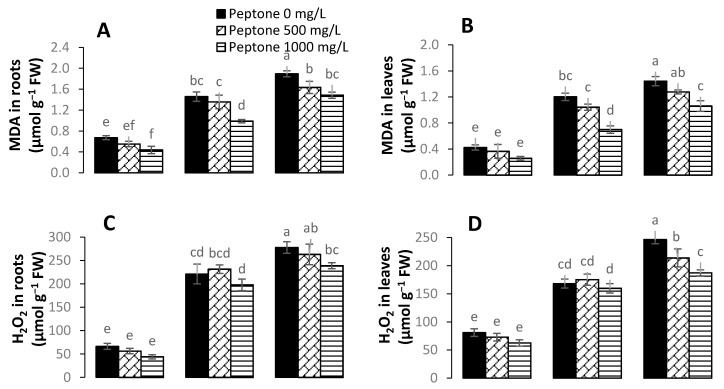

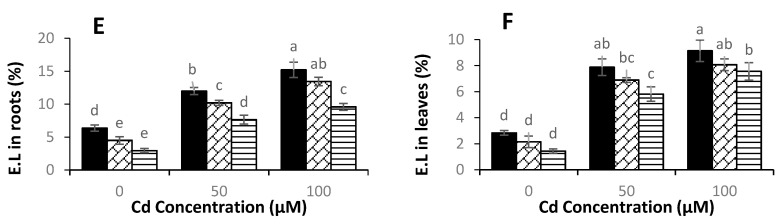
The influence of the foliar application of peptone (0, 500, and 1000 mgL^−1^) on MDA in roots (**A**), MDA in leaves (**B**), H_2_O_2_ in roots (**C**), H_2_O_2_ in leaves (**D**), EL in roots (**E**), and EL in leaves (**F**) of spinach plants grown under Cd stress. Values represent the means of four replicates with standard deviations. Letters on the bar indicate the significant differences between given treatments at *p* < 0.05.

In order to reveal the impact of peptone application on oxidative damage caused by Cd stress, contents of malondialdehyde (MDA), hydrogen peroxide (H_2_O_2_), and electrolyte leakage (EL) were recorded in spinach plants (Figure 4). The exogenous application of peptone (500 mgL^−1^) significantly decreased H_2_O_2_ content by 23%, 16%, and 11% under Cd levels of 0, 50, and 100 µM, respectively, as compared to control. The highest significant decrease of 24% in H_2_O_2_ content was noted at the higher level of both peptone (1000 mgL^−1^) and Cd (100 µM) relative to control. Plants treated with 1000 mgL^−1^ peptone at all levels of Cd (0, 50, and 100 µM) showed significant decreases in MDA content. However, the application of 500 mg/L^−1^ of peptone significantly reduced MDA content by 32%, 11%, and 11% under Cd levels of 0, 50, and 100 µM, respectively, as compared to control. Furthermore, spinach plants treated with peptone showed significant decreases in electrolyte leakage level at all Cd stress levels.

### 3.5. Activity of Antioxidant Enzymes

**Figure 5:**
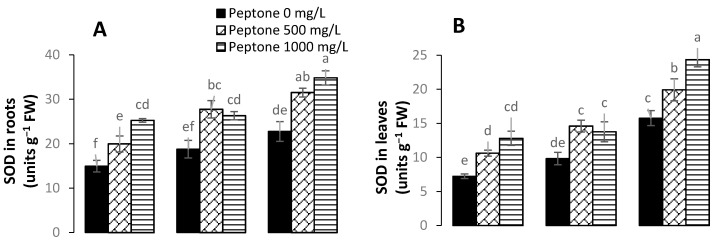

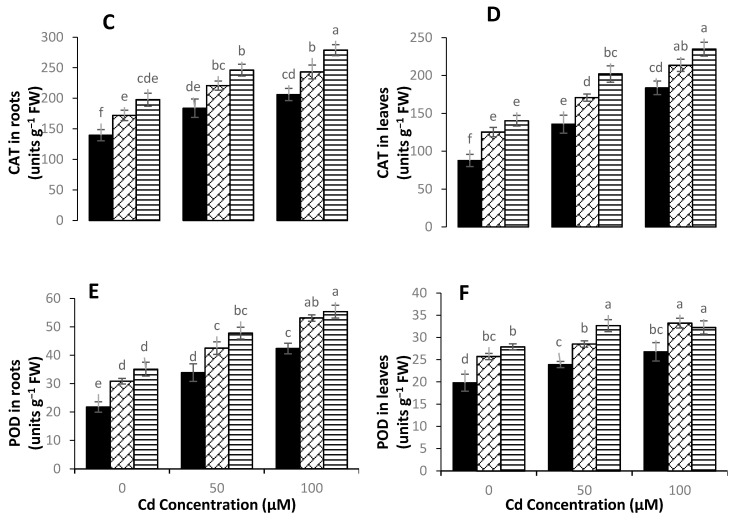
The influence of the foliar application of peptone (0, 500, and 1000 mgL^−1^) on superoxide dismutase (SOD) in roots (**A**), SOD in leaves (**B**), catalase (CAT) in roots (**C**), CAT in leaves (**D**), peroxidase (POD) in roots (**E**) and POD in leaves (**F**) of spinach plants grown under Cd stress. Values represent the means of four replicates with standard deviations. Letters on the bar indicate the significant differences between given treatments at *p* < 0.05.

In order to survive against adverse environmental conditions, plants have developed certain defense systems. Antioxidant enzymes such as superoxide dismutase (SOD), peroxidase (POD), and catalase (CAT) detoxify ROS and decrease the toxic effect of stress. The results of the current study revealed significant increases in the activity of antioxidant enzymes upon the foliar application of peptone (Figure 5). The foliar application of peptone significantly enhanced the activities of SOD and CAT (Figure 3). Moreover, significant increases of 76%, 40%, and 54% for SOD and 59%, 48%, and 27% for CAT were recorded in plants treated with 1000 mgL^−1^ of peptone. The application of 500 mgL^−1^ of peptone significantly enhanced the activity of SOD by 46%, 48%, and 26% and the activity of CAT by 42%, 25%, and 16% under the Cd stress levels of 0, 50, and 100 µM, respectively, as compared to control. The application of 500 mgL^−1^ of peptone significantly enhanced the activity of POD by 29%, 19%, and 24% under the Cd stress levels of 0, 50, and 100 µM, respectively, as compared to control.

### 3.6. Proline and Ascorbic Acid Content

**Figure 6:**
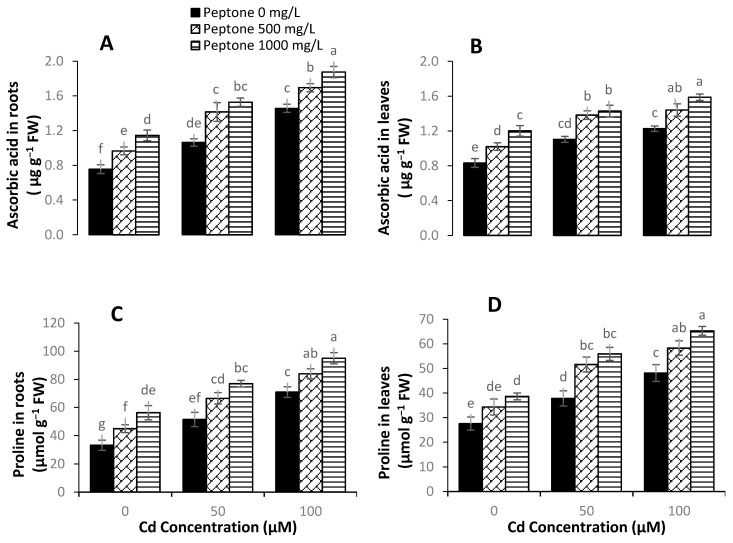
The influence of the foliar application of peptone (0, 500, and 1000 mgL^−1^) on the levels of ascorbic acid in root (**A**), ascorbic acid in leaves (**B**), proline in roots (**C**), and proline in leaves (**D**) of spinach plants grown under Cd stress. Values represent the means of four replicates with standard deviations. Letters on the bar indicate the significant differences between given treatments at *p* < 0.05.

Non-enzymatic antioxidant molecules, such as proline, could assist plants in surviving under harmful environmental conditions. The results indicated that the application of 500 mgL^−1^ of peptone significantly enhanced proline contents by 24%, 36%, and 21%, while the application of 1000 mgL^−1^ of peptone exhibited significant increases of 40%, 47%, and 35% under Cd stress levels of 0, 50, and 100 µM, respectively, as compared to control (Figure 6). The higher concentration of peptone (1000 mgL^−1^) significantly enhanced the level of ascorbic acid by 44%, 29%, and 29%, while the application of 500 mgL^−1^ of peptone resulted in significant increases of 22%, 25%, and 17% at Cd stress levels of 0, 50, and 100 µM, respectively, as compared to control.

### 3.7. Cd Concentration

**Figure 7:**
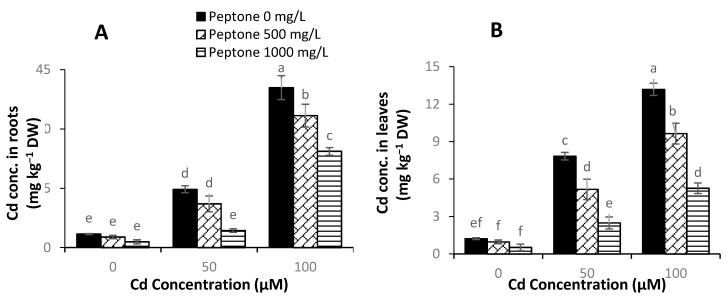
The influence of the foliar application of peptone (0, 500, and 1000 mgL^−1^) on Cd concentration in roots (**A**) and Cd concentration in leaves (**B**) of spinach plants grown under Cd stress. Values represent the means of four replicates with standard deviations. Letters on the bar indicate the significant differences between given treatments at *p* < 0.05.

The application of 500 mgL^−1^ of peptone significantly decreased the Cd concentration in plant tissues under all Cd stress levels, as compared to control. The maximum dose of peptone (1000 mL^−1^) caused the highest decreases in Cd accumulation, as compared to that in control plants (Figure 7).

## 4. Discussion

The results of the present study showed that Cd stress significantly reduced the growth and biomass of spinach plants. This decrease in the plant growth might be due to the high concentration of Cd in the plant shoot and roots as well as the higher translocation rate of Cd from plant roots to shoots. It has been well documented that the growth and biomass of different plant species were negatively affected by Cd toxicity, depending upon the type of plant species and the exposure duration to Cd. Previous studies have clearly reported these Cd-stress-induced toxic impacts on the growth traits of various plant crops [35,36]. Like the other abiotic stresses [37,38], Cd could reduce the plant growth via affecting the ultrastructure and normal functioning of plant [39]. On the other hand, the application of amino acids regulated the growth, dry weight, quality, nitrogen contents, and yield of food crops [40]. The current study showed that the increases in plant growth might be due to the direct or indirect positive effects of peptone application for spinach plants grown under Cd stress. Similarly, the application of a mixture of amino acids enhanced the growth and nutrient contents of faba bean plants in seawater-stressed environment [14]. Moreover, the foliar application of peptone enhanced the fresh and dry weight, leaf area, and growth of *Helichrysum bracteatum* L. [12]. Amino acids are rich sources of energy and carbon that help in promoting plant growth [41]. Amino acids can serve as immediate sources of nitrogen, which are usually taken up by plants more rapidly than organic nitrogen. The results of this study are in a coordination with the earlier studies conducted on lemon basil [42], basil plant [43], and *Pelargonium graveolens* L. [44]. As building blocks of proteins, amino acids also play key roles in the regulation of metabolism and nitrogen storage.

Previous studies reported the adverse effects of environmental stresses on the growth, chlorophyl content, and gas exchange attributes of different plant species [45,46,47,48,49,50,51,52,53]. Similarly, in the current study, Cd stress decreased the chlorophyll content and gas exchange parameters in spinach plants. These decreases might be due to the change in chloroplast structure under Cd stress [54]. Cd stress results in a reduction in chlorophyll contents by causing lipid peroxidation, which results in a disturbance in the structure and function of the thylakoid membrane [55]. Additionally, the decrease in chlorophyll content under Cd stress might be due to the overproduction of ROS, a naturally reactive compound that damages pigments and other biomolecules. On the other hand, in the present study, the foliar application of peptone reduced the damaging effects of Cd stress on chlorophyll content of spinach plants. Previous studies also reported the positive roles of amino acids in increasing the photosynthetic pigments in *Foeniculum vulgare* L. [56], *Salvia farinacea* L. [57], and faba bean [13]. In the same way, three different concentrations of peptone (250, 500, and 1000 mg/L^−1^) enhanced chlorophyl *a* and chlorophyl *b* contents in *Helichrysum bracteatum* L. [12]. It is likely that the application of peptone, being a source of free amino acids, reduced Cd toxicity in spinach plants as amino acids play key roles in stress signaling and secondary metabolism [58]. In the current study, the foliar spray of peptone enhanced the gas exchange parameters such as stomatal conductance and photosynthetic rate in spinach plants under Cd stress. These results were in agreement with that previously reported [35]. Previous reports have revealed that the environmental stresses considerably induce oxidative stress as well as the levels of electrolyte leakage (EL) and malondialdehyde (MDA) in different plant species, causing damages in plant tissues [59,60,61,62,63,64,65,66]. Similarly, in the current study, spinach plants also exhibited higher levels of EL and MDA in the absence of peptone treatment. The increased levels of EL and MDA in the roots and shoots of spinach plants indicated the Cd-stress-induced oxidative stress. However, in the present study, the foliar spray of peptone decreased EL and MDA contents in plant shoots and roots as compared to control. This decrease in the level of oxidative stress might be due to the protective role of peptone against lipid peroxidation in spinach plants grown in a Cd-stressed environment. The antioxidant enzymes play a key role in ROS scavenging in order to enhance the plant capability to cope with stress conditions [38,67]. It was reported that peptone application provided an optimum concentration of radially available free amino acid molecules, which in turn reduced the free radicals associated with enhanced osmoprotection [68]. Similar results were previously reported in rice plants grown under Cd stress [35]. In addition, the availability of amino acids (e.g., glutamate) from a biostimulant (e.g., peptone) may alleviate the oxidative stress via increasing the synthesis of stress-responsive amino acids such as arginine and proline [22].

The current study also revealed decreases in the activity of antioxidant enzymes in spinach plants grown under Cd stress. Such decreases in antioxidant enzymes activity might be due to the higher concentration of Cd in plants, which in turn increased the levels of EL and MDA. Additionally, the increase or decrease in the activity of antioxidant enzymes depends upon the level of metal stress and plant species [69,70]. In the present study, the application of peptone promoted the antioxidant enzymes activities. The enhanced activities of SOD, POD, and CAT due to peptone application might be due to the lower concentrations of Cd in these plants. Furthermore, the mobility of Cd in the active plant parts might be reduced due to the amino acid ability to form a complex with Cd, resulting in decreases in Cd toxicity [71]. This enhancement in enzymes activity promoted spinach plants’ tolerance to Cd stress via reducing the levels of EL and MDA in plant tissues.

The regulation of osmotic adjustment substances such as proline could maintain the cell membrane integrity and osmotic balance in plants [72]. The results of the current study revealed an increase in the contents of ascorbic acid and proline. The increase in the concentration of osmotic adjustment substances in plants suppresses the toxic effects of Cd stress [73]. In the present study, the application of peptone decreased Cd contents in the shoot and roots of spinach. This decrease in Cd contents might be because peptone application increased the amino acids’ availability and modulated the intracellular environment, which in turn restricted the Cd uptake and translocation [22].

## 5. Conclusions

Cd stress exerts negative impacts on the growth and physio-biochemical attributes of spinach plants. Foliar spray of peptone significantly promoted the plant growth and biomass. It also enhanced the activity of antioxidant enzymes, which promoted the plants’ defense against Cd stress. The application of peptone also reduced the levels of H_2_O_2_ and MDA. The amino acids have the ability to bind with Cd and immobilize it. The use of peptone application against metal stress is not well-studied, hence further field studies on other food crops are suggested.

## Figures and Tables

**Table 1:** The characteristics of the soil used in the experiment of the present study.

Soil Characteristics	Values
Texture	Sandy clay
Sand %	48
Silt %	16
Clay %	36
pH	7.65
EC dS m^−1^	0.811
**Soluble ions**	
Ca^2+^ mmol L^−1^	7.71
Na mmol L^−1^	9.46
K mmol L^−1^	1.34
**Metal concentrations**	
Cd mg kg^−1^	0.83
Pb mg kg^−1^	6.64
Zn mg kg^−1^	4.93
Mn mg kg^−1^	6.37
Fe mg kg^−1^	50.34
